# Advances in the Diagnosis, Monitoring, and Progression of Oral Cancer through Saliva: An Update

**DOI:** 10.1155/2022/2739869

**Published:** 2022-10-25

**Authors:** Jesús Rodríguez-Molinero, Blanca del Carmen Migueláñez-Medrán, Esther Delgado-Somolinos, Carmen Martín Carreras-Presas, Antonio Francisco López-Sánchez

**Affiliations:** ^1^Department of Nursing and Stomatology. Faculty of Health Sciences, King Juan Carlos University, 28922 Alcorcón. Madrid, Spain; ^2^Adult's Dentistry Department. Oral Medicine, European University of Madrid, 28670 Villaviciosa de Odón. Madrid., Spain

## Abstract

The early detection of cancer, and in particular oral cancer, has been a priority objective of study in recent years. Saliva has been proposed as an easy-to-obtain means of providing the necessary information to diagnose malignant lesions in the oral cavity, since it can be obtained very easily and completely noninvasively. There are a number of molecules, known as biomarkers, which may be involved in the malignant transformation of oral lesions, and which have different natures. The involvement of proteins (“proteomics”), metabolites (“metabolomics”), and even certain genes in the structural changes of altered tissue has been investigated in order to establish validated parameters for the early diagnosis of oral cancer. In addition, the development of new analytical assay methods that can reduce costs and obtain better results in terms of sensitivity and specificity has been a key point in recent research in this field. Even though there are numerous biomarkers with results showing high sensitivity and specificity, there is still a need for more studies, with a larger sample and with analytical methods that can constitute a real advance in time and cost. Although salivary biomarkers are a promising new diagnostic tool for oral cancer, for the moment they do not replace biopsy as the “gold standard”.

## 1. Introduction

Oral cancer is the sixth most common cancer worldwide [1]. Tobacco use, alcohol consumption, and overinfection with some oncogenic papillomavirus (HPV) serotypes have been shown to be the main risk factors for developing oral cancer. In addition, other factors such as sun exposure and chronic mucosal irritation are also considered factors that may increase the risk of developing oral cancer [2]. Moreover, diet has also been suggested as a protective or risk factor for oral cancer according to Rodríguez-Molinero et al. [3].

Performing an early diagnosis is necessary to obtain better prognosis and survival rates, in addition to improving the quality of life of patients [4].

Intraoral systematic examination of high risk of malignancy areas is a key point for the diagnosis of oral cancer. Nowadays, the gold standard for the diagnosis of oral cancer is still the biopsy and histopathological examination, but lately, the scientific community has been trying to find other tools to be able to perform early diagnosis of oral cancer in a less invasive way, introducing salivary diagnostics of biomarkers that could be used as screening and monitoring prognosis of the disease [4–6].

According to the FDA-NIH Biomarker Working Group, a biomarker is defined as a characteristic that is measured as an indicator of normal biological processes, pathogenic processes, or responses to an exposure or intervention. Biomarkers should be tested and validated in terms of specificity and sensitivity. Thus, biomarkers could be diagnostic, monitoring, pharmacodynamic/response biomarkers, predictive, and prognostic [7].

Saliva is a complex secretion, consisting of 98% water and organic and inorganic particles [8]. Its secretion is regulated by the autonomic nervous system. Whole saliva or oral fluid refers to the liquid made up of salivary gland secretion, crevicular fluid, epithelial cells shed from the oral mucosa, and food debris [9]. Other components may include DNA, proteins, metabolites, RNA, and microbiota which have been proposed as biomarkers of certain diseases. Collecting saliva is easy, noninvasive, and well tolerated by patients [10]. As a result, point-of-care device technology has been a breakthrough in the detection of oral and systemic diseases such as viral (including SARS-CoV-2), bacterial or fungal infections, and cancer or genetic disorders [11, 12]. Therefore, saliva can be considered a suitable, convenient, and extraordinary diagnostic biofluid [13].

Researchers have classified these biomarkers into fields or boxes of knowledge, under the term of “OMICS” (or “SALIVAOMICS”), including genome and epigenome (the study of genes and methylation), transcriptomics (the study of mRNA within cells or organisms), metabolomics (the study of global metabolite profiles in a system), lipidomics (the study of the lipidic profile), proteomics (the study of proteins), and microbiota (the study of microbiology) [14].

The purpose of this review is to highlight the recent advancements in the OMIC disciplines concerning oral cancer diagnostic biomarkers.

## 2. Material and Methods

A search was carried out using the databases PubMed, Medline and SCOPUS with keywords “saliva”; “oral cancer”; “cancer biomarkers”; “oral cancer detection”; “mRNA”; “proteomics”; and “metabolomics” in various combinations, using the Boolean operators “AND” and “OR”. To make sure the subject to be reviewed was up to date, the search was limited to articles published in the last 5 years, more specifically between January 2016 and July 2021. The search was limited to articles published in English, and related to studies carried out with saliva in humans. Studies that analyzed oropharyngeal cancer without specifying location were eliminated. Review articles, opinions, letters to the editor, editorials, news, clinical cases, in vitro studies, and any clinical trials not based on a case-control study were excluded.

## 3. Results

**Figure 1:**
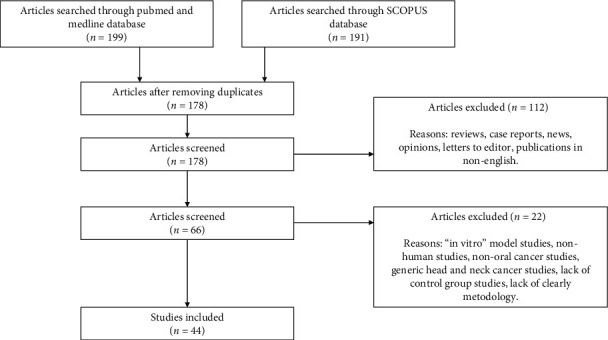
Literature search sequence.

The initial search resulted in 390 articles. A first selection reduced this number to 178 articles with possible interest. Subsequently, the titles were preliminarily assessed, and unpublished articles in English were excluded as well as reviews, letters to the editor, news, opinions, and clinical cases, resulting in a total of 66 articles available for use. After a more exhaustive assessment of the abstract, it was decided to eliminate 19 articles due to the following reasons: (1) studies in “in vitro” models, (2) studies in nonhuman models, (3) studies that made reference to other types of cancers, (4) studies that referred generically to head and neck cancers, (5) studies that did not have a control group, and (6) studies that did not establish a precise methodology. Finally, 47 studies were selected. The search sequence is reflected in Figure 1.

## 4. Discussion

The collection of saliva can be considered a useful and convenient method for noninvasive diagnosis of certain diseases, within this oral cancer [15]. In most of the articles reviewed, saliva collection took place in the mornings [16–32]. Salivary secretion varies throughout the day and is influenced by factors such as temperature, the different seasons of the year, the individual's hydration status, and the medication they take [10]. Not to forget the different types of diet that also alters salivary composition. The same subject is likely to have different rates of salivary secretion at different times of the day [15].

Regarding the sample collection, a certain preference for collecting unstimulated saliva stands out: only two studies used stimulated saliva [29, 33], and only one indicated that the patient should spit [34]. The preferred method of collection is “unstimulated whole saliva”, although it must be kept in mind that an “unstimulated” state of saliva is never going to be completely unstimulated; it is impossible to standardize an unstimulated collection of saliva, as small involuntary movements and stimuli can alter the secretion of the same [15]. The collection of saliva can even be influenced by the duration of collection: too short or too long collection periods can result in unreliable amounts [35]. Furthermore, to standardize sample collection conditions, patients should be instructed on how to properly perform the saliva collection, without eating or drinking 90 minutes prior to collection and making the least possible mouth movements [36, 37]. The “whole saliva” collection method includes, in addition to the salivary secretion itself, the presence of fluids, bacteria, and cellular debris. The main advantage of this collection method is that it is very easy to perform, mainly because it is a noninvasive and stress-free procedure for the patient [15]. Collection of saliva in ice-chilled vials is recommended to slow down the activity of hydrolytic enzymes present in saliva. In addition, it is advisable to collect saliva in vials containing protease inhibitors (EDTA (ethylenediaminetetraacetic acid), PMSF (phenylmethanesulfonylfluoride), soybean trypsin inhibitor, and E-64 for example to inhibit proteolysis when protein purification is desired. These compounds do not interfere with biomarker analysis [15]. When it is not possible to collect in vials with ice, sodium azide can be added to the sample to delay bacterial growth. It is important to keep in mind that this compound is an inhibitor of those enzymes that contain heme-groups such as peroxidases, and therefore may interfere with some laboratory tests, such as the ELISA test [15].

The conserving of the sample is recommended in liquid nitrogen at -80°C, as performed by most of the authors consulted. At higher storage temperatures, protein degradation may occur, however, Hashimoto et al., Rhai et al., and Khyani et al. stored them at -20°C. [16, 26, 38] When the sample is to be processed, it should be thawed as quickly as possible. Centrifugation is performed after collection in order to discard bacteria and cellular debris, as bacteria are rich in hydrolytic enzymes that could break down salivary biomarkers. Centrifugation is another aspect that differs greatly among the analyzed literature. It has been observed in the literature how the centrifugal force varies with values between 2600 and 10000 × *g*, and in other cases, the value used is the revolutions per minute (rpm) ranging between 1200 and 11000 rpm. The ideal situation would be that all the salivary biomarkers have the same collection, conservation, and processing method [15].

### 4.1. Analysis of the Main Groups of Biomarkers

#### 4.1.1. Proteomics

There are many salivary biomarkers currently under investigation, the field of proteomics being the one presenting the greatest number of studies. Higher levels of tumor necrosis factor (TNF-*α*) have been found in oral squamous cell carcinoma (OSCC) than in controls, and even in premalignant lesions [17], significant differences are noted between patients with early-stage OSCC and controls [39]. Dikova et al. indicate in their research, carried out in Spain, that TNF-*α* (among other markers) may be useful in the discrimination and distinguishing between patients with OSCC and patients with oral leukoplakia and healthy subjects [40].

Various studies analyze the prognostic value of interleukins (IL) interpreted as tumor biomarkers in cases of OSCC. Lee et al. state that, as is the case with TNF-*α*, there are significant differences in the levels of IL-1*β*, IL-6, and IL-8 in saliva between those patients with OSCC and those subjects that are healthy [39]. In contrast, another study did not find significantly elevated levels of IL-6 in all groups; however, elevated levels of IL-8 are found [38]. Singh et al. state that they found elevated levels of IL-1*β* and IL-8 in all stages [25]. This theory is supported by other authors, asserting that IL-6 and IL-8 may have diagnostic utility to discriminate between OSCC and healthy patients, noting that IL-6 is elevated in cases of stages T3 and T4 and those stages in which there is cervical metastasis [40].

Other possible biomarkers such as metalloproteinases 1 and 3 (MMP1, MMP3) peptidyl arginine deiminase type 1 (PADI1), tenascin-C (TNC), and cystatin-A (CSTA) have also shown significant levels in patients with OSCC [41]. Hsiao et al. and Chang et al. detected elevated levels of MMP1 in patients with OSCC, while these levels were not detectable in healthy subjects allowing to establish a possible biomarker that can discriminate healthy subjects from patients [42, 43]. Furthermore, they show that MMP1 levels may correlate with tumor progression [43]. MMP9 has shown significantly higher levels in patients with OSCC than in those with premalignant oral lesions (OPMD) as well as those in the control group [29, 44]. It was also found that the values of epidermal growth-factor receptor (EGFR) [45], solute carrier family 3 member (SLC3A2), and S100 calcium-binding protein A2 (S100A2) [46] are increased in saliva in those patients suffering from OSCC. In addition, some cases demonstrated altered levels of certain biomarkers such as CD44, S100 calcium-binding protein A7 (S1000A7), and S100 calcium-binding protein P (S100P) even in cases of leukoplakia with epithelial dysplasia [22].  Table 1.

#### 4.1.2. Metabolomics

The salivary metabolic profile has often been called the “body's mirror” because it provides an overview of significantly modified metabolites from aberrant enzymatic regulation, captures oncometabolites that arise from metabolic re-arrangement, and highlights altered pathways during metabolic reprogramming [56–58]. More than 100 metabolites have been described to show alteration with malignant progression of OSCC. Some mentioned are choline, carnitine, lactate, glutamate, sialic acid, histidine, polyamines, pipecolic acid, trimethylamine N-oxide, and S-adenosylmethionine [58–61]. To study, it can be more or less complicated depending on the evaluation system used. Mass spectrometry (MS) is a very useful tool for proteomics and metabolomics and has become commonplace in biomedical research due to its deep coverage of various molecular species. However, many of the MS systems require a rather complex pretreatment time for biological samples, especially when working with a large number of samples. But the development of methods such as CPSI-MS (Conductive polymer spray ionization mass spectrometry), capable of analyzing a wide spectrum of metabolites in a few seconds, allows for studies in large population groups. Song et al. suggest that the combination of CPSI-MS and ML (machine learning) constitutes a feasible tool for accurate and automatic diagnosis of OSCC. In their study of 373 patients, 175 whom suffered from OSCC; slight metabolic changes were found, starting with the aminoacyl t-RNA biosynthesis, arginine biosynthesis, and arginine metabolism, lysine degradation, histidine metabolism, and proline metabolism during the precancerous stage. These findings provide potential clinical markers to indicate OSCC tumorigenesis. Furthermore, they demonstrate that CPSO-MS is a promising environmental mass spectrometry tool, with cost-effective performance in monitoring hundreds of salivary metabolites without the need for laborious sample pretreatment, as is the case with the other procedures [58].

In another study using capillary electrophoresis time-of-flight mass spectrometry on a sample of 24 patients with OSCC and 44 healthy subjects, they found that both pipecolic acid and S-adenosylmethionine showed significantly higher levels in patients with OSCC [59], while Ohshima et al. used the same method and obtained significant levels of taurine, valine, leucine, isoleucine, choline, cadaverine, and tryptophan, in addition to the presence of 3-hydroxybuyric acid in the metabolism of patients with OSCC [62].

Another group of metabolites studied as a possible tumor marker is that of monosaccharides. Serum-L-fucose has been reported to be elevated in various cancers, including breast cancer, colorectal cancer, cervical cancer, lung cancer, and brain tumors [23]. Fucose is a monosaccharide component of many N- and O-linked glycans and glycolipids produced by mammalian cells. It has been observed that remodeling of cell surface glycoproteins and glycolipids by modifying oligosaccharide structures is associated with the biological behavior or tumor cells. These being mechanisms through which fucose can be associated with cancer and inflammation. There are several studies carried out on serum, but so far, only one in saliva. In this research, the levels of L-fucose in the saliva of 30 OSCC patients were studied, comparing them with healthy subjects and with patients with oral potentially malignant disorders (OPMD). In the group of OSCC patients, the levels of L-fucose were significantly elevated, which confirms the fact that fucosylated glycans are overexpressed in tumor cells, and that they are continuously secreted in saliva [23].

Among the biomarkers suggested for the study of oral cancer, we also find volatile organic compounds (VOCs) present in saliva, although there is scarce literature on the subject. The technique used for its assessment is gas chromatography-mass spectrometry. Lipid peroxidation of polyunsaturated fatty acids present in cell membranes, reactions which are triggered by oxidative stress, is the mechanism considered as the main mechanism in the formation of these differentiated VOCs associated with diseases. Aro et al. conducted a study in Poland on 30 subjects of which five had oral cancer. They selected a total of 32 VOCs, and after eliminating those possibly due to oral bacteria growth, they saw a significant result in a total of five VOCs (E-2-octenal, heptanoic acid, octanoic acid, E-2-nonenal, nonanoic acid, and 9-undecenoic acid), derived from linoleic, palmitoleic, and oleic acid, increasing in patients with oral cancer, although Aro et al. emphasize that the sample studied was small [13]. VOCs have also been studied by Shigeyama et al. in Japan, performing a comparative analysis of salivary profiles of VOCs on 12 patients in OSCC. Altered values of 12 VOCs were found, some due to a decrease with healthy patients (Ethanol, 2-Pentanone, Phenol and Hexadecanoic acid); others disappeared (Undecane, 1-Octanol, Butyrolactone and Benzyl alcohol), and four were newly appeared (3- Heptanone, 1,3-Butanediol, 1,2-Pentanediol and 1-Hexadecanol) [63].  Table 2.

#### 4.1.3. Genetic Biomarkers

OSCC biomarkers using salivary RNA have been analyzed, including messenger RNA (mRNA), microRNAs (miRNA), and salivary DNA. It was long believed that RNA was rapidly degraded by enzymes (ribonucleases) in saliva, but it is now known that RNA is protected by its degradation by exosomes [64]. Exosomes are extracellular vesicles contained in a lipid bilayer between 50-150 nm in diameter that contain molecules such as mRNA, microRNA and proteins. Exosomes can be secreted from cells or organs into bodily fluids (blood, urine, saliva.) These circulating exosomes contain potential biomarkers. However, there are two important problems: the first being that techniques used for processing can determine differences in the composition of exosomes, and the second being that the composition of salivary exosomes can vary due to external factors such as alcohol consumption, tobacco, or HPV infections, so these factors need to be taken into consideration in studies [20]. Detection is performed using microarray analysis and quantitative real time PCR (qRTPCR), the latter method more sensitive. Results to measure the validity of transcripts are made using ROC (receiver operating characteristic) curve analysis, determining the adequacy of specificity and sensitivity.

*(1) Messenger RNA (mRNA)*. mRNA can be isolated in saliva and can verify an increase or decrease of mRNA transcripts in patients with OSCC. The objective of the current research is to find transcripts or combinations of them, with their modification showing enough significance to be able to become biomarkers with adequate sensitivity and specificity for the detection of OSCC. Most authors use similar detection methods, while Han et al. propose a new detection method: SERS (surface-enhanced Raman Spectroscopy). They describe this method as the most suitable for its application in a health center, finding concentrations of S100P (S100 calcium-binding protein P) mRNA tripled in patients with OSCC [19]. However, the study is limited to only 3 patients and 3 controls. Another study analyzed the decrease in monoamine oxidase B (MAOB), NGFI-A-binding protein 2 (NAB2), collagen type III alpha 1 (COL3A1), CYP27A1, NPIPB4, and sialic acid acetylesterase (SIAE) in patients with OSCC. Here, they found that the greatest decrease in mRNA levels in MAOB and NAB2 occurs in patients younger than 60 years, with an AUC (area under the curve) of 0.91, while it was not significant in patients over 60 years [65].

Just as we can appreciate differences of expression depending on age, in another study it was observed that there may also be differences by gender in certain markers. The expression of leucine zipper downregulated in cancer-1 (LDOC1) was increased in women with OSCC, while what could be a biomarker amongst men was the low level of LDOC1 [34]. Another suggestion is the significant expression of nuclear undecaprenyl pyrophosphate synthase 1 (NUS1) and reticulocalbin 1 (RCN1) in OSCC patients compared to healthy individuals. NUS1 has been found to be effective in detecting cases of OSCC, but with a high level of false positives in healthy patients. When combined with RCN1, the negative predictive value improves [21]. The role of ciliogenesis and planar polarity effector 1 (CPLANE1) has been analyzed in patients with OSCC, patients with potentially malignant disorders (leukoplakia and lichen planus without distinguishing between these pathologies, and healthy subjects. Results show that CPLANE1 could be used as a salivary biomarker for early detection, as it detected OSCC with a sensitivity of 0.8 and a specificity of 0.925. However, performing the analysis by age, it was seen that in the group over 60 years there was a higher number of false positives [66].

*(2) MicroRNA*. MicroRNAs (miRNAs) are a subclass of short RNAs (19-23 nucleotides), involved in the regulation of various physiological processes, playing an important role in tumor progression and oncogenesis by negatively regulating gene expression at a post-transcriptional level, and intervening in the processes of cell growth, apoptosis, differentiation, motility, and immunity [64]. One of the mechanisms responsible for the reduction or loss or miRNA expression is through epigenetic mechanisms due to DNA methylation at the genetic regulation sites of microRNA [55]. miRNAs are stable in the blood, and when circulating they can be indicative of cancer phenotype, thus used as noninvasive tools for cancer staging and prognosis. Many miRNAs can regulate a gene, and multiple genes can be regulated by a single miRNA [67].

miR-let-7a-5p and miR-3928 have been proposed as possible biomarkers of OSCC as these show a significant decrease in patients with OSCC [68]. Salazar et al. also found a significant decrease in miR-124-3p in OSCC, and an increase in miR-146a-5p, although in his sample he did not specify the number of patients with OSCC [67].

He et al. look for markers that appear increased in cases of OSCC, such as miR-24-3p, which is significantly elevated in OSCC patients (by analyzing the ROC curve, the AUC of miR24-3p was 0.7838, indicating that it allows for the significant distinguishing of patients and healthy subjects in with 64.4% sensitivity and 80% specificity [20]. In all of the RNA analysis articles from the past 5 years, a striking observation is that none of them analyze two equal factors or increase the number of the study sample. A number of different study elements are presented making it difficult to choose one or a group of biomarkers that have appropriate levels of specificity and sensitivity to be used as an early diagnosis.

*(3) DNA-Based Markers*. Aberrant DNA-methylation can be detected in saliva. The methylation of p16INK4a, RASSF1A, TIMP3 and PCQAP/MED15 has been studied in patients with OSCC compared to controls. Its relationship with risk factors such as tobacco, alcohol, and betel nut has been analyzed, and it can be concluded that these factors are considered promoters of hyper methylation of the aforementioned genes, and that they can be found in OSCC patients [69]. It has been hypothesized that there exists a possibility DNA methylation of miRNA-encoding loci (mgmiRs) can be used for detection of cancer in tissue and its correlation in saliva. Being a genomic technique based on DNA it is less dependent on sample processing techniques than techniques based on the detection of mRNA or proteins. In addition, DNA methylation occurs in early stages of cancer, so it can be used for early detection. A combination of the 7 (mgmiRs) analyzed in saliva allowed the detection of 76.9% of the OSCC [70].  Table 3.

## 5. Limitations

Despite promising results of many of the included articles, there still exist limitations making it difficult to use salivary biomarkers as valid tools in the early diagnosis of oral cancer. There is a lack of standardization in the management of samples, with regard to all collection, processing, and storage. One of the first variables that may affect the levels of salivary biomarkers is the selection of the saliva collection method, the time of collection and the type of saliva collected. The vast majority of studies use the “whole saliva”, unstimulated and collected during the morning. This method is simple and inexpensive in addition to not requiring special elements for its collection—unlike methods that stimulate the secretion of saliva from specific glands. Few articles use stimulated saliva despite research suggesting that there is greater precision in the detection of cancer-related biomarkers [71, 72]. However, we found that the time of collection is not precisely specified, or very little data is provided regarding this. Adding to this fact, some studies do not specify their population inclusion and exclusion criteria. These are important factors; the subjects serving as cases and controls must be perfectly defined to avoid false positives or false negatives. Although it has been observed that the optimal storage temperature of saliva is -80°C, there are studies using lower temperatures or do not provide data in this regard. Per now, there is no research pointing to which centrifugation speed is the best, and so there is great variability between studies that may influence the detection of biomarkers [73]. Detection methods are diverse and complex. Test systems that can replace ELISA and RT-PCR have been investigated to improve sensitivity and cost, but further research is required to validate the results.

There remains a lack of standardization in the quantification of salivary biomarker levels that identify healthy individuals and patients with oral cancer or precancerous lesions. This could perhaps be explained by a wide interindividual variability associated with factors such as gender, race, and habits [72]. In addition, it should be taken into account that other pathologies can alter the levels of the biomarkers themselves, such as periodontal disease, inflammatory diseases and trauma or fungal infections, which must be recorded in the medical history [74]. The use of an isolated biomarker obtains worse results in terms of diagnostic precision, with lower values of sensitivity and specificity, than when several are combined. It is better to use them simultaneously, which implies studying several biomarkers, demanding a greater investment in terms of costs [75].

## 6. Conclusions

Despite the limitations of the articles, salivary biomarkers constitute a promising new diagnostic tool. For the time being, the use of biomarkers as a diagnostic tool cannot replace the biopsy as the “gold standard”, and more resources are needed for its widespread use in prevention and diagnosis of oral cancer to be implemented. Although there are numerous biomarkers of different nature with results showing high sensitivity and specificity, more studies with larger sample sizes and analytical methods that can constitute a real advance in time and cost are necessary.

## Figures and Tables

**Table 1:** Proteomic salivary biomarkers.

Author	Country	Number of samples	Biomarker category	Biomarker	Saliva collection time	Saliva collection method	Sample processing	Test method	Results
Azeem et al. [28]	India	96 OSCC patients / 96 tobacco chewers without lesions/96 healthy patients	Protein	PBSA FSA	9 am-12 pm	Unstimulated whole saliva	Centrifuged at 3000 rpm/15 min.	Spectrophotometric	Salivary PBSA and FSA are significantly raised in tobacco chewers and OSCC patients.
Chang et al. [43]	Taiwan	269 OSCC patients /578 OPMD/313 healthy patients	Protein	MMP1	—	Unstimulated whole saliva	Centrifuged at 3000 × *g*/15 min/4°C. Stored at -80°C.	ELISA	MMP1 levels in saliva distinguish between OSCC patients and healthy controls. MMP1 levels in OSCC patients was strongly correlated with tumor progression.
Chi et al. [41]	China	30 OSCC patients /30 healthy patients	Protein	ANXA2, CA2, CD44, CSTA, DSG3, EGFR, ENO1, FLNA, GANAB, HSPA5, IL-6, ISG15, KRT18, LGALS3BPMMP1, MMP2, MMP3, MYO5A, OASL, PADI1, S100A2, SERPINE1, SPP1, STAT1, TIMP1, TNC, TYMP, ULBP2, WARS, YWHAB	Morning and afternoon	Unstimulated	Centrifuged at 3000 × *g*/15 min/4 °C.	SISCAPA-MRM	MMP1, PADI1, TNC, CSTA and MMP3 showed significant levels in OSCC patients and a high level of disease discrimination.
Cohen et al. [47]	USA	150 OSCC patients /150 healthy patients	Protein	SolCD44, TP	—	Unstimulated saliva	Transport on ice Storage at -80°C	Sandwich ELISA	Association of solCD44 with worse progression-free survival data.
Csősz et al. [30]	Hungary	20 OSCC patients/17 healthy patients	Protein	Proteomic profile	Between 9 a.m. and 11 a.m.	Unstimulated saliva	Centrifuged at 4100 × *g*/15 min/4 °C. Stored -70°C	LC-MS analysis/ELISA	None of the studied proteins turned to be a potential biomarker in OSCC.
Csősz et al. [31]	Hungary	55 OSCC patients/52 healthy patients	Protein	IL-1*α*, IL-1*β*, IL-6, IL-8, TNF-*α*, VEGF catalase, profilin-1, S100A9, CD59, galectin-3-bindig protein, CD44, thioredoxin and keratin-19	Between 9 a.m. and 11 a.m.	Unstimulated saliva	Centrifuged at 4.000 × g/15 min/4 °C. Stored at -70°C.	LC-MS analysis	The validation of IL-6, S100A9 and thioredoxin revealed the potential utility of combination of IL-6 and S100A9.
Dikova et al. [40]	Spain	66 OSCC patients / 33 OHL/33 OPVL/25 healthy patients	Protein	IL-1*α*, IL-6, IL-8, IP-10, MCP-1, TNF-*α*, HCC-1, and PF-4	—	Unstimulated whole saliva	Centrifuged at 3,000 × *g*/15 min/4 °C. Stored at -80°C.	ELISA multiplex immunoassay	L-6, IL-8, TNF-*α*, HCC-1, discriminate between OSCC and healthy patients. IL-6, IL-8, and PF-4 discriminate OSCC from OLK without specifying the type.
Ghallab et al. [44]	Egypt	15 early stage OSCC patients/15 OPMD patients/15 healthy patients	Protein	Chemerin, MMP-9 in serum and saliva	Morning	Unstimulated whole saliva	Centrifuged at 10000 × *g*/2 min. 2 min/10,000 × *g* Stored at−80°C.	ELISA	Serum and salivary levels of chemerin and MMP-9 in patients with OSCC were significantly higher than OPMLs and control group. Patients with OPMLs showed also elevated profiles for serum and salivary chemerin and MMP-9 com- pared to control group.
Heawchaiyaphum et al. [48]	Thailand	92 OSCC patients/83 healthy patients	Protein	HP PRDX-2 ZAG	—	—	Centrifuged at 1200 rpm /15 min/4°C. Stored at –80°C.	LC-MS/MS Western blot	PRDX-2 and ZAG upregulated in OSCCc HP upregulated in OSCC. IGHA2 downregulated in OSCC.
Hsiao et al. [49]	Taiwan	9 OSCC patients / 5 healthy patients	Protein	MMP1	—	Unstimulated whole saliva	Centrifuged at 3000 × *g*/15 min/4°C. Sored at-80°C.	ImmunoMALDI analysis	Elevated MMP1 levels in saliva in 7 of 9 OSCC patients. MMP1 was not detectable in healthy subjects.
Ishikawa et al. [50]	Japan	39 OC patients /31 healthy patients	Protein	Cornulin, Alfa2 macroglobulin-like protein, hemoglobin subunit beta, Ig kapa chain V-II region V kappa 167, Kininogen-1 Transmembrane protease serine 11D	—	Unstimulated saliva	Centrifuged at 2600 × g/15 min/4°C.	LC-MS/MS	Cornulin lower in patiens with OC. The combination of all the analized biomarkers have a high accuracy for differenciating between patients with oral cancer and control.
Kang *et al*. [33]	China	20 OSCC patients/20 OSCC patients from OLK/20 OSCC patients from OLP/20 OLK/20 OLP/20 healthy patients	Protein	KLK5 uPA	—	Stimulated for 10 minutes	Centrifuged at 10000 × g/10 min/4 °C. Stored at -80°C.	ELISA	The results suggested that the combination of KLK5 and uPA could represent a potential biomarker for determining the malignant transformation of OLK or OLP into OSCC.
Khyani et al. [38]	Pakistan	35 OPMD/35 OSCC patients 35 healthy patients	Cytokine	IL-6, IL-8	—	Unstimulated saliva	Centrifuged at 4500 rpm/15 min/4°C. Stored at -20°C.	ELISA	IL-6 levels not significantly elevated in all groups. IL-8 levels significantly elevated in all groups.
Ko et al. [51]	Taiwan	86 OSCC pateients/35 healthy patients	Protein	AKR1B10	—	Unstimulated whole saliva	Centrifuged at 1000 × g/10 min/4 °C. Stored at -80°C.	ELISA	AKR1B10 levels were significantly higher in the patients with OSCC than in the normal controls. Higher salivary AKR1B10 levels were significantly associated with larger tumor size, more advanced clinical stage, and areca quid chewing habit.
Lee et al. [39]	Taiwan	41 OSCC patients/ 24 healthy patients	Cytokine	EGF, Eotaxin, G-CSF, IFN- g, GRO, IL-10, IL-1*α*, IL-1*β*, IL-6, IL-8, IP-10, MIP-1*β*, TNF-*α*, VEGF	—	Unstimulated saliva	Centrifuged at 3000 rpm/20 min/4°C. Stored at -80°C.	Multiplex (Luminex bead-based)	Eotaxin, IL-1*β*, IL-6, IL-8, MIP-1*β*, TNF-*α*: Significant differences between early stage OSCC patients and healthy patients.
Lin et al. [52]	Poland	5 OSCC patients/5 healthy patients	Protein	Global proteomic profile	10 a.m. to 12 p.m	Unstimulated whole saliva	Centrifuged at 2600 g/20 min/4°C. Stored at -80°C.	LC-MS/MS	Oral cancer may have varied pathological effects on the saliva proteome.
Michailidou et al. [32]	Greece	34 OSCC patients/20 OLK patients/31 helathy patients	Protein	IL-1B, IL-8, OAZ and SAT	Morning	Unstimulated saliva	Centrifuged at 2600 × *g*/15 min/4 °C. Stored at -80°C.	RT-PCR	SAT and IL-8 mRNAs have good discriminatory ability for oral squamous cell carcinoma patients Only 81.3% sensitivity and 73.9% specificity in the diagnosis of oral squamous cell carcinoma at an early stage.
Peisker et al. [29]	Germany	30 OSCC patients/30 healthy controls	Protein	MMP-9	Between 7 -8 am	Stimulated saliva	Centrifuged at 1000 × g/20 °C/2 min.	ELISA	The elevation of salivary levels of MMP-9 may be a useful adjunctive diagnostic tool for detection of OSCC.
Rai et al. [26]	India	25 OSCC patients /24 healthy patients	Cytokines	IL-6 IL-8 TNF-*α* IFN-*γ* GM-CSF	9.00 am to11.00 am	Unstimulated whole saliva	Centrifuged at 9300 g/5 min. Stored at -20°C.	Bio-Plex pro™ human cytokines assay	The largest amount of S. *Anginosus,* V. *Parvula*, P. *Endodontalis* and P. *Anaerobius* may help to the development of OSCC via increased expression of proinflamatory cytokines.
Seyedmajidi et al. [53]	Iran	20 OSCC patients/20 healthy patients	Protein	Soluble CD44 in serum and saliva	10 a.m. to 12 p.m.	Unstimulated whole saliva	Centrifuged at 2000 and 11000 rpm/10 min. Stored at -80°C.	ELISA	There was no statistically significant difference in serum and saliva solCD44 level between the patient and control groups. There was no significant correlation between the solCD44 level in each patient and control group in serum.
Shan et al. [46]	China	20 OSCC patients/20 OPMD patients/20 healthy patients	Protein	SLC3A2 S100A2 IL1RN	Morning	Unstimulated whole saliva	Centrifuged at 2600 × g/15 min/4°C. Stored at -80°C.	iTRAQ y ELISA	SLC3A2 and S100A2 levels were significantly increased in the OSCC group compared to healthy controls and OPMD. IL1RN levels were significantly decreased in the OSCC group compared to healthy controls and OPMD.
Singh et al. [25]	India	31 OSCC TNM I-II and 27 27 TNM III-IV patients/30 OPMD patients/29 post treatment patients/ 42 healthy patients	Protein	IL-1*β* IL-8 LGALS3BP	Morning	Unstimulated	Centrifuged at 1000 × g/20 min/2-8°C. Stored at -80°C.	ELISA	IL-1*β* and IL-8 were significantly elevated in all stages OSCC patients. LGALS3BP was elevated in early OSCC patients.
Sivadasan et al. [22]	India	15 dysplasticOLK/15 LP 15 OSCC N_0_ 15 OSCC N+ patients/15 healthy patients	Protein	93 proteins	Morning	Unstimulated	Centrifuged at 2000 rpm/4°C/10 min and centrifuged at 14000 rpm. Storage at -80°C.	LC-MS and ELISA	CD44, S1000A7 and S100P levels significantly altered in patients with leukoplakia with dysplasia and OSCC.
Wang et al. [54]	China	79 OSCC patients/31 OPMD patients 80 healthy patients	Protein	SNCG	—	Unstimulated	Centrifuged at 1000 × g/2 min/room temperature. Stored at -80°C.	ELISA	SNCG expression is related with medium and low degrees of cell differentiation in OSCC.
Yu et al. [55]	Taiwan	103 low risk OPMD/130 high riskOPMD/ 131 OSCC patients/ 96 healthy patients	Protein	ANXA2 HSPA5 KNG1 MMP1	—	Unstimulated whole saliva	Centrifuged at 3000 × g/15 min/4°C. Stored at -80°C.	LC-MRM-MS	The four-protein panel could be used to diagnose OSCC with an 80% specificity.
Zanotti et al. [45]	Italy	63 OSCC patients/ 60 healthy patients	Protein	EGFR	—	Unstimulated	Centrifuged 13000 rpm/3 min. Storage at -80°C.	Sandwich ELISA	Higher EGFR concentration in saliva in OSCC patients. Higher EGFR levels in patients with higher T category. Worse prognosis in patients with higher EGFR levels.
Zheng et al. [27]	China	112 OSCC patients 30 OPML 60 healthy subjects	Protein	Naa10p CEA	9.00 am to 11.00 am	Unstimulated whole saliva	Saliva: Centrifuged at 800 × g/2 min/room temperature. Blood: Centrifuged at 1200 × g/10 min/room temperature. Stored at -80 °C.	ELISA	Combined diagnosis of Naa10p and CEA significantly increased both sensitivity and specificity. Combined diagnosis with Naa10p and CEA used like salivary biomarkers resulted more sensitive than serum.

FSA: free sialic acid; PBSA: salivary protein-bound sialic acid; OHL: oral homogeneous leukoplakia; OPVL: oral proliferative verrucous leukoplakia; OPMD: oral potentially malignant disorder; OLK: oral leukoplakia; OLP: oral lichen planus; OC: oral cancer; OSCC: oral squamous cell carcinoma; ELISA: enzyme-linked immunosorbent assay; LC-MRM-MS: liquid chromatography-multiple reaction monitoring-mass spectrometry; SISCAPA-MRM: stable isotope standards and capture by anti-peptide antibodies coupled with multiple reaction monitoring-mass spectrometry; RT-PCR: real-time polymerase chain reaction; iTRAQ: isobaric tags for relative and absolute quantitation, SNCG: synuclein-gamma; EGFR: epidermal growth factor receptor; CEA: carcinoembryonic antigen; KLK5: kallikrein 5; Upa: urokinase-type plasminogen activator; MMP: metalloproteinase; TNF-*α*: tumour necrosis factor-*α*: IL: interleukine; KNG1: kininogen 1, HSPA5: heat shock 70 kDa protein 5; ANXA2: annexin 2; S1OOA2: S100 calcium-binding protein A2; IFN-*γ*: interferon-*γ*; GM-CSF: granulocyte macrophage colony-stimulating factor;.

**Table 2:** Metabolic salivary biomarkers.

Author	Country	Number of samples	Biomarker category	Saliva collection time	Saliva collection method	Sample processing	Test method	Results
Lohavanichbutr et al. [60]	USA	101 OSCC patients/58 OPC patients/35 healthy patients	Metabolites	—	Unstimulated	Centrifuged at 1300 × g/10 min/4 °C. Stored at -80°C.	LC-MS	The levels of two metabolites (glycine and proline) to be significantly different between OCC and controls but did not find any appreciable differences in metabolite levels between OPC and controls or between OCC with and without nodal metastasis. Four metabolites, glycine, proline, citrulline, and ornithine were associated with early-stage OCC.
Monedeiro et al. [18]	Poland	5 OSCC patients/15 healthy patients	VOCs	Between 8 and 12 AM	Unstimulated	Stored at -80°C.	HS-SPME + GC-MS analysis	1-octen-3-ol, hexanoic acid, E-2-octenal, heptanoic acid, octanoic acid, E-2-nonenal, nonanoic acid, 2,4-decadienal and 9-undecenoic acid. Obtained overall accuracy was 90%. Oral cancer cases were predicted with 100% of sensitivity and specificity.
Shigeyama et al. [63]	Japan	24 OSCC patients/50 healthy patients	VOCs	For at least 1.5 h after meals for multiple (1–3) time periods up to surgery after hospitalization	Unstimulated	Stored at −80°C.	TFME method + GC-MS	12 potential BM of oral cancer detection: -decreased levels: Ethanol, 2-Pentanone, phenol, Hexadecanoic acid. -disappeared levels: Undecane, 1-octanol, Butyrolactone, benzyl alcohol. -new VOCs production: 3-Heptanone, 1,3-Butanediol, 1,2-Pentanediol, 1-Hexadecanol.
Song et al. [58]	China	249 (124 OPMD, 125 OSCC)/124 healthy patients	Metabolites	Multiple	Unstimulated	Centrifuged at 5000 rpm/3 min. Stored at -80°C.	CPSI-MS	Putrescine, cadaverine, thymidine, adenosine, 5-aminopentoate, hippuric acid, phosphocholine, glucose, serine, adrenic acid showed changes in their levels (increasing or decreasing) during malignant progression from health to OSCC.
Ohshima et al. [62]	Japan	22 OSCC patients/21 healthy patients	Metabolites	Early morning 8 : 00 a.m.	Unstimulated	Centrifuged at 2600 × g/15 min/4 °C, and spun for a further 20 min.	CE-TOF/MS	Choline, BBCAs, urea, and 3-hydroxybutyric acid were statistically significant in the detection of OSCC.
Ishikawa et al. [59]	Japan	24 oral cancer patients/44 healthy patients	Metabolites	08 : 00 am–12:00	Unstimulated whole saliva.	Centrifuged at 9100 × g for at least 2.5 h/4°C Stored at −80°C	CE-TOF/MS	Combination of Pipecolato and S-adenosylmethionine yielded a high area under receiver operating characteristic curves for discriminating oral cancers from controls.
Sharma et al. [23]	India	55 (30 OPMD y 25 OSCC) patients/30 healthy patients	Monosaccharids	Early morning	Unstimulated	Centrifuged at - Stored at -80°C.	Spectrophotometry	Significantly higher L-fucose levels in OPML and OSCC groups in saliva.

OSCC: oral squamous cell carcinoma; OPMD: oral potentially malignant disorders; VOCs: volatile organic compounds; HS-SPME+GC-MS: headspace-solid phase microextraction and analyzed using gaschromatography-mass spectrometry; TFME method+GC: thin-film microextraction method and chromatographic analysis with mass spectrometric; LC-MS: Liquid chromatography–mass spectrometry; CPSI-MS: conductive polymer spray ionization mass spectrometry; CE-TOF/MS: capillary electrophoresis time-of-flight mass spectrometry; BCAA: metabolites in the branched chain amino acids cycle.

**Table 3:** Genetic salivary biomarkers.

Author	Country	Number of samples	Biomarker category	Saliva collection time	Saliva collection method	Sample processing	Test method	Results
Cao et al. [70]	USA	95 OSCC patients/92 healtht patients	DNA: mgmiRs 9-1 124-1, 124-3, 129, 137, 148a	_	Unstimulated whole saliva.	Stored at -80°C.	qMS-PCR	The combination of the 7 markers in saliva detects 71.4% of OSCC.
FadhilI et al. [68]	Australia	150 HNSCC patients/80 healthy patients	miRNA: -miR-let-7a-5p -miR-3928		Unstimulated whole saliva.	Samples were immediately stored in dry ice and sent for processing in the laboratory.	qRT-PCR analysis,	This study concluded that salivary miR-let-7a-5p and miR-3928 has the potential to be novel noninvasive biomarkers for early detection and prognosis of HNSCC. miR-let-7a-5p significantly influenced by cancer staging and lymph node metastasis.
Han et al. [19]	USA	3 OSCC patients/3 healtht patients	mRNA S100P	Between 6 am and 12 am	Unstimulated whole saliva	Centrifuged at2600g/15 min/4°C.	SERS-based assay	The concentration of S100P mRNA in OSCC patients was three times higher than in the healthy group.
He et al. [20]	China	49 OSCC patients/ 14 healthy patients 30 OSCC undergoing surgery (tissue sample)	miRNA: miR-24-3p	Morning (9.00 am to 12.00 pm)	Unstimulated whole saliva	Centrifuged at 2600 × g/30 min/4°C. Stored at-80°C.	qRT-PCR microarray analysis	RT-PCR confirmed a significant increase of miR-24-3p in 45 OSCC compared to 10 healthy subjects miR-24-3p was expressed higher in OSCC.
Liu et al. [34]	Taiwan	53 OSCC patients/ 43 healthy patients	mRNA: - LDOC1	—	Spitting	Centrifuged at 2000 g/10 min/4°C. stored at −80°C.	qRT- PCR	qRT-PCR data revealed no significant difference between all the OSCC subjects and all the normal subjects. LDOC1 expression levels by gender, there was a significant difference between male and female OSCC subjects compared to normal males and females.
Liyanage et al. [69]	Sri-Lanka- Australia	54 oral cavity cancer patients 34 oropharyngeal cancer/ 60 healthy patients	DNA: - P16INK4a - RASSF1A - TIMP3 - PCQAP - MED15	—	—	Centrifuged at 1200 × g/10 min/4°C.	MS-PCR	RASSF1A, TIMP3 and PCQAP/MED15 appeared hypermethylated in OC and OPC compared to healthy controls.
Oh et al. [65]	Korea	33 OSCC patients/ 34 healthy patients	mRNA: - NAB2 - CYP27A1 - NPIPB4 - MAOB - SIAE - COL3A1	—	Unstimulated whole saliva	Centrifuged at 5600 rpm/15 min/4°C. Stored at -80°C.	qRT-PCR	-levels of mRNA de MAOB, NAB2, COL3A1, CYP27A1, NPIPB4, SIAE decreased in OSCC. -mayor disminución de niveles de mRNA en MAOB y NAB2. -combination of CYP27A1 + SIAE better for early detection of OSCC. -combination of MAOB+NAB2 better predictive values for younger than 60 with OSCC.
Salazar-Ruales et al. [67]	Ecuador	108 HNSCC	miRNA: - miR-122-5p - miR-92a-3p - miR-124- 3p - miR-205-5p - miR-146a-5p	—	Unstimulated whole saliva	—	PCR Array	miR-124- 3p decreased in OSCC and miR-146a-5p increased in OSCC.
Ueda et al. [21]	Japan	41 OSCC patients/ 10 healthy patients	mRNA: C1S, ELFN1, NUS1, RCN1, FCN1, HDHD1, GSTO1, KIAA1257, RSAD2	Morning	Unstimulated whole saliva	Stored at -80 °C.	qRT-PCR	Significant expression of RCN1 and NUS1 in OSCC patients versus healthy patients. NUS1: False + in healthy patients. RCN1: High predictive value - high.
Ueda et al. [66]	Japan	42 OSCC patients/ 42 healthy patients	CPLANE1	Morning preferred	Unstimulated whole saliva	Centrifuged at 14000 × g/2 min. Stored at -20°C.	qRT-PCR	CPLANE1 expression levels were significantly higher in OSCC patients than in healthy and OPMDs patients.

AUC: area under curve; COL3A1: collagen type iii, alpha1; CYP27A1: cytochrome P450, family27, subfamilyA, polipeptide1; ddPCR: droplet digital PCR; HNSCC: head and neck squamous cell carcinoma; LCMS/MS: liquid chromatography-mass spectrometry; MAOB: monoaminooxidasa B; miRNA: micro RNA; MS-PCR: methylation-specific polymerase chain reaction; NAB2: NGFI-abinding protein 2; NPIPB4: nuclear pore complex interacting protein family member B4; OSCC: oral squamous cell carcinoma; OPMD: oral potentially malignant disorders; qMS-PCR: quantitave methylation-specific PCR; qRT- PCR: cuantitative real time PCR; ROC: receiver operating characteristics; RT-PCR: real time PCR; SERS: surfaced-enhanced Raman spectroscopy; S100P: S100 calcium-binding protein P; SIAE: sialic acid transferase; -: not specified.

## Data Availability

The figures, tables and all data used to support the findings of this study are included within the article.
